# Habitat-specific differences alter traditional biogeographic patterns of life history in a climate-change induced range expansion

**DOI:** 10.1371/journal.pone.0176263

**Published:** 2017-05-04

**Authors:** Megan E. Riley, Blaine D. Griffen

**Affiliations:** 1 Department of Biological Sciences, University of South Carolina, Columbia, SC, United States of America; 2 School of the Earth, Ocean, and Environment, Marine Science Program, University of South Carolina, Columbia, SC, United States of America; Universitat Trier, GERMANY

## Abstract

Range shifts and expansions resulting from global climate change have the potential to create novel communities with unique plant-animal interactions. Organisms expanding their range into novel biotic and abiotic environments may encounter selection pressures that alter traditional biogeographic patterns of life history traits. Here, we used field surveys to examine latitudinal patterns of life history traits in a broadly distributed ectotherm (mangrove tree crab *Aratus pisonii*) that has recently experienced a climate change-induced range expansion into a novel habitat type. Additionally, we conducted laboratory and field experiments to investigate characteristics associated with these life history traits (e.g. fecundity, offspring quality, and potential selection pressures). We compared these characteristics in native mangrove habitats in which the species has historically dwelled and novel salt marsh habitats into which the species has recently expanded its range. Consistent with traditional biogeographic concepts (i.e. Bergmann’s clines), size at maturity and mean body size of reproductive females increased with latitude within the native habitat. However, they decreased significantly in novel habitats at the highest latitudes of the species’ range, which was consistent with habitat-specific differences in both biotic (predation) and abiotic (temperature) selection pressures. Although initial maternal investment (egg volume and weight) did not differ between habitats, fecundity was lower in novel habitats as a result of differences in size at reproduction. Offspring quality, as measured by larval starvation resistance, was likewise diminished in novel habitats relative to native habitats. These differences in offspring quality may have enduring consequences for species success and persistence in novel habitats. Life history characteristics such as those investigated here are fundamental organismal traits; consequently, understanding the potential impacts of climate change responses on latitudinal patterns of these traits is key to understanding climate change impacts on natural systems.

## Introduction

In recent decades, changes in global climate trends have led to radical alterations in natural ecosystems. A number of systems have experienced losses in biodiversity as well as changes in the composition and dynamics of communities [1–2], both of which are predicted to continue in the future [3–4]. One of the most common responses to a warming climate is a shift in the range or distribution of species. Because the geographic ranges of most plants and animals are limited to some extent by climatic factors (i.e. temperature, light, precipitation) [3], changes in global climate trends have caused many species to shift or expand their distributions to include higher altitudes and more poleward latitudes [5–6]. For example, species whose ranges are constrained by the cold are expanding their distributions poleward in response to warming temperatures, with terrestrial species expanding at an average rate of 16.9 km each decade [5] and marine species expanding at an average rate of 72 km each decade [6]. Such range expansions have been widely documented in an extensive array of taxonomic groups, including insects [7–8], plants [9], mammals [10], and marine invertebrates [11].

Because different taxonomic groups display variation in their sensitivity and response to climate changes, species often shift their ranges at different speeds [12]. These differences in the speed of range expansion have the potential to lead to spatial mismatches between previously interacting species [12–14]. When rapidly expanding species expand their range faster than their neighbors, this process creates novel ecosystems with unique community assemblages and plant-animal interactions [3, 12]. The resulting novel communities often represent a suite of new selection pressures that may prompt rapid changes in plastic life history traits in range-expanding organisms [15–17]. Because many organisms already exist across historically broad ranges in which they display latitudinal clines in life history traits, selection pressures associated with their range expansion may alter existing biogeographic life history patterns [17].

For example, body size is among the most fundamental life history characteristics of an organism, influencing key traits such as individual fitness and population dynamics [18]. Biologists have long documented variations in both body size and size at maturity across latitude, including increases in body size and size at maturity with latitude (see [19] for a list of reviews). Bergmann [20] originally addressed this topic by noting that endotherms in cooler temperatures (i.e. higher latitudes) tend to have larger body sizes than those in warmer temperatures (i.e. lower latitudes) as a result of thermoregulatory mechanisms. Bergmann’s clines are now commonly used to describe intraspecific patterns in the body size of both endotherms and ectotherms across latitude [21–22]. Although there is no definitive consensus on the mechanisms underlying body size clines in ectotherms [19], factors such as temperature, resource availability, and predation are strong drivers of body size patterns [22–24].

Selection pressures associated with range expansions may influence biogeographic phenomena such as Bergmann clines, as well as trends in other physiological and reproductive characteristics [17]. Here, we examine the potential for range expansion into a novel habitat to alter life history traits within the context of existing latitudinal life history clines using the model organism *Aratus pisonii* (mangrove tree crab). It is one of numerous species whose recent climate-induced range expansion has led to a spatial mismatch with a habitat-forming plant species [25]. Within its native habitat, this neotropical mangrove crab exists in close association with several mangrove species, including red, black, and white mangroves (*Rhizophora mangle*, *Avicennia germinans*, and *Laguncularia racemosa*, respectively). In particular, the red mangrove *R*. *mangle* provides both food and shelter for this arboreal species [26]. Although mangroves in Florida have experienced a substantial recent poleward expansion along the Atlantic coast due to a decrease in the frequency of extreme cold events [9], the climate change-induced poleward expansion of *A*. *pisonii* (estimated at 62 km/decade in the last century [25]) has outpaced that of its native habitat and the species has established itself in novel vegetation in temperate salt marshes [25].

We identified latitudinal patterns of life history traits (female size at maturity and average size at reproduction) of *A*. *pisonii* across a broad (13.73°) latitudinal gradient that included its native mangrove habitat and novel salt marsh vegetation. Additionally, for a representative of each habitat type (native mangrove and novel mixed marsh) we compared the proportion of females actively reproducing at multiple time points as well as fecundity and metrics of maternal investment (egg volume and weight) and offspring quality (larval starvation resistance). Finally, because selection pressures such as predation may vary between habitats, and this may influence the life history traits of interest here (e.g. [27–28]), we used simple metrics designed to assess the ability of individuals to utilize the vegetation structure in novel habitats as a potential predator evasion strategy, and compared size-specific predation risk in both native and novel habitats.

## Methods

### Determination of latitudinal life history trends

**Fig 1:**
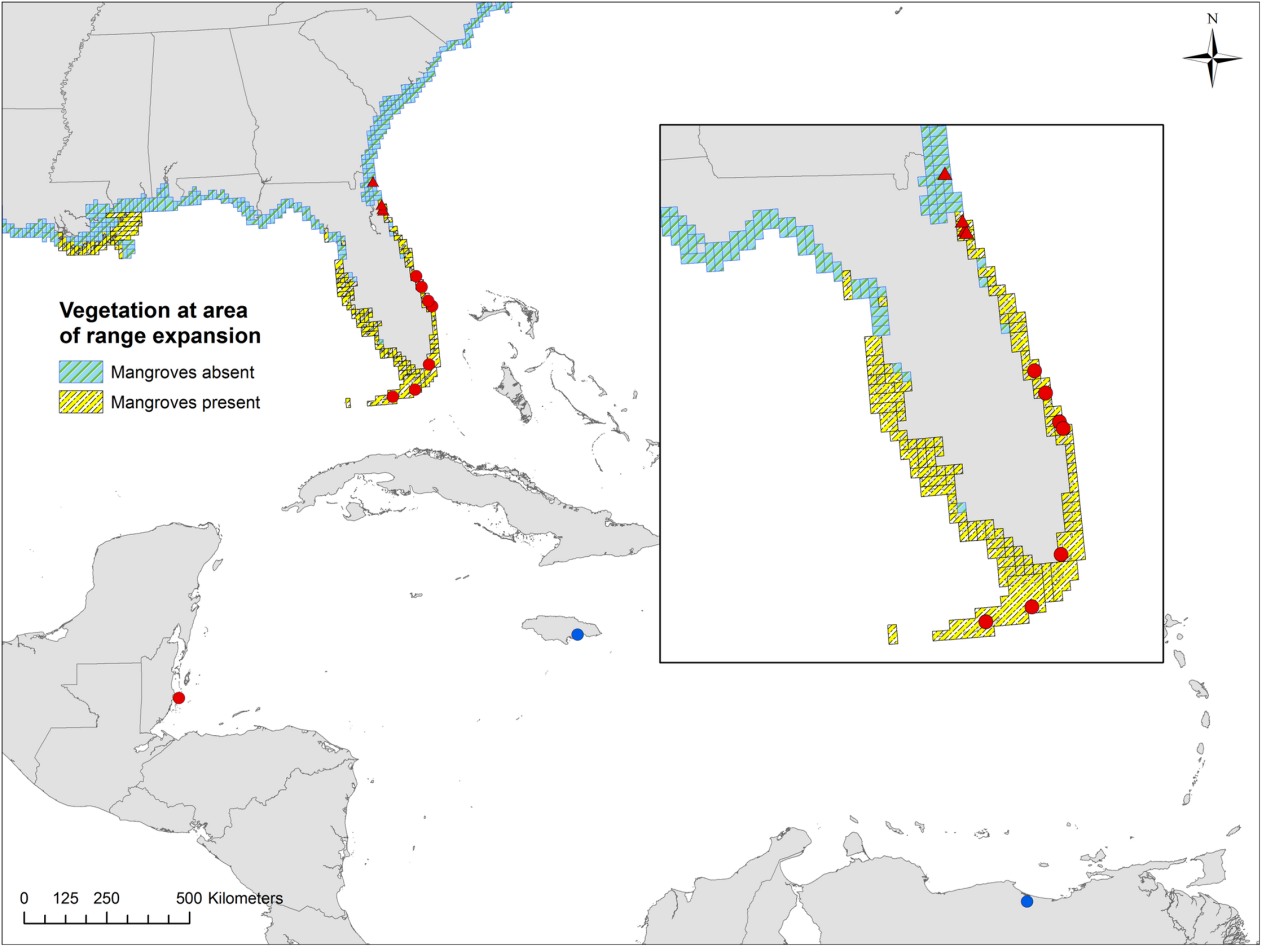
Map exhibiting the geographic distribution of sampling sites. Circle symbols represent mangrove habitats; triangle symbols represent marsh habitats. Red symbols represent sites sampled by the authors; blue symbols represent sites for which data was established from the literature [31–32]. Map was prepared with ESRI ArcGIS v10 software using mangrove distribution data from [33].

In order to determine latitudinal patterns of body size and size at maturity, we conducted population surveys at 12 sites spanning a 13.73° latitudinal range in the northern hemisphere (Table 1, Fig 1). All field research was conducted with permission from the appropriate authority, which included the Florida Department of Environmental Protection (Permit Numbers: 04031310, 04221420), United States National Park Service (Permit Number: BISC-2012-SCI-0022), Florida Fish and Wildlife Conservation Commission (License Number: SAL-13-1497-SR), and Belize Fisheries Department (Reference Number: GEN/FIS/15/04/2013 (54) Vol. IX). No protected species were disturbed or sampled during research activities in the field.

**Table 1:** Description of sites where *Aratus pisonii* body size distribution surveys were conducted.

Survey Site	Habitat	Latitude (°N)	Sample size
Laguna de Tacarigua National Park, Venezuela^1^	Mangrove	10.87	[31]
Twin Cays, Belize	Mangrove	16.78	132
Port Royal Mangroves, Jamaica^2^	Mangrove	17.93	[32]
Kemp Channel, FL, USA	Mangrove	24.66	128
Keys Marine Lab, FL, USA	Mangrove	24.83	101
Biscayne Bay National Park, FL, USA	Mangrove	25.46	89
Hobe Sound National Wildlife Refuge, FL, USA	Mangrove	27.09	137
St. Lucie Inlet State Park, FL, USA	Mangrove	27.14	104
Avalon State Park, FL, USA	Mangrove	27.55	127
Sebastian Inlet State Park, FL, USA	Mangrove	27.85	115
Fort Matanzas, FL, USA	Mixed Marsh	29.73	98
Devil's Elbow, FL, USA	Mixed Marsh	29.75	86
Anastasia State Park, FL, USA	Mixed Marsh	29.88	140
Big Talbot Island State Park, FL, USA	Salt Marsh	30.51	115

Field surveys performed by the authors were supplemented by two sites from the literature

^1^Laguna de Tacarigua National Park, Venezuela [31] and

^2^Port Royal Mangroves, Jamaica [32].

Of the 12 sites surveyed, eight were pure mangrove habitats, three were hybrid mangrove-salt marsh habitats (hereafter referred to as mixed marsh), and one was a pure salt marsh habitat. The number of mixed and pure salt marsh sites surveyed was constrained by the extent of hybrid marsh sites and the limited distribution of *A*. *pisonii* in pure salt marsh habitats. The term “mixed marsh” used here refers to a site that is dominated by salt marsh cordgrass *Spartina alterniflora* with isolated dwarf mangrove trees occasionally interspersed among the cordgrass. However, because mangroves themselves have only recently begun expanding their range into these salt marsh habitats [9, 29], these dwarf trees are younger and smaller than those found further south in mature mangrove forests. As a result, the mangrove trees in mixed marshes are highly connected to the surrounding salt marsh, and their small size and isolated nature likely do not lend themselves to the same microhabitat creation that has been documented in mature mangrove forests (e.g. [30]). These mixed marshes therefore more closely resemble salt marshes than mangrove forests.

At each site, crabs (mean sample size ± SE = 112.36 ± 6.93 at each site) were randomly collected during daytime high tides, at which time they are the most active foraging above the intertidal zone [34]. In the mangroves, crabs were collected by hand from the lower canopy and prop roots above the water line. In the salt marsh, crabs were collected by hand from exposed cordgrass. We measured their carapace width (CW) to the nearest tenth of a millimeter, identified their sex, and determined the reproductive status (gravid or not gravid) of females. We considered size at maturity to be the smallest gravid female collected at each site, consistent with previous methods of determining size at maturity in the species [31–32]. In order to expand our latitudinal coverage of size at maturity data, we also included published reports of size at maturity from two additional sites in mangrove habitats [31–32], which both used the same method for determining size at maturity. We subsequently included size at maturity values from these literature sources in the same statistical analysis as the values established by our surveys. Thus, we maintained methodological consistency by considering size at maturity to be the smallest gravid female collected at each site, rather than employing alternative methods for determining size at maturity (e.g. [35]).

In order to determine the influence of latitude and habitat type on (1) size at maturity and (2) average size of ovigerous females, we used separate general linear models for each response variable. Latitude and habitat type were included as explanatory variables. The interaction term between these variables was initially considered, but it was not significant for the model of size at maturity (P = 0.844) or the model of average size of ovigerous females (P = 0.331). Therefore, the interaction was removed to simplify both models.

### Comparison of body size distributions and reproductive effort at representative sites

We conducted additional surveys throughout the summer reproductive peak (June, July, and August 2013) to further investigate body size distributions and reproductive effort. Due to logistical constraints, these additional surveys were restricted to two representative sites: a representative mature mangrove forest (Avalon State Park, Fort Pierce, FL, 27.55°N) and a representative mixed marsh (Anastasia State Park, St. Augustine, FL, 29.87°N), which were chosen after careful consideration of several factors. Both sites (1) exemplify the prominent characteristics of each habitat type, (2) occur at relatively similar latitudes but are neither the northernmost nor southernmost site surveyed for each habitat type, and (3) initial surveys demonstrated that body size patterns at the two sites were consistent with the overall latitudinal patterns described here, which were established based on 12 sampling sites and two additional sites identified from the literature [31–32].

At each of these representative sites, we collected female crabs (mean sample size ± SE = 57.17 ± 4.14 at each time point) as described previously. This sampling (mangrove: June (n = 41), July (n = 62), August (n = 53), marsh: June (n = 55), July (n = 58), August (n = 69)) increased our total sample size for the representative mangrove and marsh sites to n = 156 and n = 182, respectively. For each individual, we measured CW to the nearest tenth of a millimeter and determined their reproductive status (ovigerous or not ovigerous). Because *A*. *pisonii* carry multiple broods each year, we took advantage of *A*. *pisonii*’s reproductive lunar synchronization [32] and conducted all monthly surveys in the week preceding the full moon to capture periods of maximum reproductive effort for both populations. From these surveys, we compared body size distribution patterns of reproductively mature and gravid females, as well as the proportion of females that were gravid at each of the three monthly samples.

Body size data was analyzed using a Wilcoxon rank-sum test and Fisher’s F-test in order to compare mean size and size variability between the two populations, respectively. Additionally, Hartigan’s dip test for unimodality [36] was calculated to determine the body size distribution (unimodal, bimodal, etc.) of both reproductively mature (i.e. above the site-specific size at maturity but not ovigerous) and gravid females in the population in order to elucidate whether a single cohort or multiple cohorts were reproducing simultaneously in each habitat type. The proportion of gravid females in each habitat type was determined for each monthly survey by dividing the total number of gravid females by the total number of females larger than the site-specific size at maturity from each monthly sampling effort. This data was analyzed using a generalized linear model with a binomial distribution and month and habitat as categorical explanatory variables.

### Comparison of maternal investment, offspring quality, and fecundity at representative sites

In order to determine offspring quality, we collected ovigerous females independent of the stage of their brood’s embryonic development from the representative mangrove (n = 21) and mixed marsh (n = 18) sites described in the previous section and transported them live to the Smithsonian Marine Station in Fort Pierce, FL. Crabs were maintained at ambient temperature with a natural light: dark cycle in individual plastic aquaria (22.8 cm L x 15.2 cm W x 16.5 cm H) containing approximately 200–300 mL of filtered seawater (salinity ~31 ppt) and a piece of plastic mesh (20 cm x 4.5 cm) that provided a substrate on which crabs could exit the water. Crabs were fed plant material (*S*. *alterniflora* for crabs collected from the mixed marsh habitat and *R*. *mangle* for crabs collected from the mangrove habitat) *ad libitum*. Both food and water were changed every 48 hours. Although there is no published literature documenting the food sources or dietary preferences of *A*. *pisonii* in marsh habitats, M. Riley conducted dissections of individuals collected from the marsh and observed plant material with striations characteristic of *S*. *alterniflora* in the crabs’ gut contents. In addition, individuals offered only *S*. *alterniflora* in captivity for up to two weeks produced frass, which further demonstrated that *S*. *alterniflora* is a potential food source for the species. Moreover, all crabs included in the offspring quality experiment were gravid upon collection from the field, so the diet crabs were fed in captivity did not influence reproductive investment or offspring quality in the brood examined.

A small portion (<1/3) of each female’s egg mass was gently removed from the pleopods with forceps within 24 hours of collection from the field, placed into filtered sea water (~31 ppt), and visually examined under a Leica M205C dissecting microscope at 80X to determine egg developmental stage. Eggs were categorized as non-eyed (mangrove n = 10, marsh n = 15) or eyed (mangrove n = 11, marsh n = 3) based on visual inspection of each female’s brood. For females carrying recently extruded, non-eyed eggs, which are reflective of initial maternal investment in the quantity of egg yolk, a subsample of the removed eggs (n = 20–30) was photographed using a Jenoptik ProgRes C14+ camera attached to the Leica M205C dissecting microscope and ProgRes CapturePro v2.8.0 software. Egg size was then determined by randomly selecting 10 eggs from the photographed egg mass and measuring the circular area of each egg with imageJ software. Capitalizing on the spherical nature of *A*. *pisonii* eggs, these measurements of circular area were used to determine egg volume and averaged to provide the mean egg size for each female. The impact of maternal habitat on mean egg volume was determined using a general linear model.

Other than the non-eyed eggs sampled for egg size determination, the remainder of each female’s brood (whether non-eyed or eyed eggs), was allowed to remain on the pleopods and continue normal development. Crabs were monitored twice daily for larval hatching. Twelve newly hatched larvae from each brood (mangrove n = 21 and mixed marsh n = 18 as described previously) were randomly selected and pipetted into individual Kimble borosilicate glass culture tubes (15 mm x 85 mm) with ~80 mL of 0.2 μm filtered seawater. Water was partially changed and larval mortality was monitored daily to determine starvation resistance (days) as a proxy for maternal energy provisioning. The impact of habitat on larval starvation resistance was determined using a mixed effects Cox model, with unique maternal identity included as a random effect, thus preventing pseudoreplication by accounting for the twelve larvae collected from each individual female while simultaneously focusing on the importance of maternal habitat on larval starvation resistance.

In order to determine egg weight and fecundity, additional ovigerous females (n = 22 per site) were collected from the same two representative sites and placed immediately upon ice. Eggs were removed from the pleopods of each female, egg stage (eyed/noneyed) was determined, and crabs with recently extruded, non-eyed eggs were dissected to ensure that they were post-vitellogenic (i.e. all eggs were extruded). A subset of eggs was taken from 15 randomly selected crabs from each habitat type and counted under a dissecting microscope. These subsets were dried at 65°C for 48 hours and weighed. We determined the relationship between egg count and dry egg mass using a linear regression; the slope of this relationship indicates the average dry mass of a single egg [37]. We then measured the total brood dry weight of the ovigerous individuals collected from both habitats and used the total brood dry weight and the average dry mass of a single egg to calculate the total fecundity of individuals of varying sizes collected from both habitats (n = 22 per site). The impact of habitat on individual egg weight was analyzed with a general linear model with dry mass of the subset of eggs as the response variable and egg count and habitat as explanatory variables. Fecundity was analyzed using a general linear model with maternal body size (CW) and habitat as explanatory variables.

### Predator avoidance capability and comparison of size-specific predation potential

In order to investigate the shift in body size distribution to smaller individuals in the mixed marsh habitats revealed by our population surveys (see Results), we conducted two simple assessments. First, we assessed the ability of large *A*. *pisonii* collected from mangrove habitats to climb the habitat structure available in novel salt marsh habitats. Arboreality is one of the most important predator evasion techniques employed by this species in native mangrove systems [34], where they are preyed upon by larger crabs, mangrove snappers (*Lutjanus griseus)*, ibises, and raccoons in the lower canopy and on the mud surface [26,32].

The goal of this experiment was to determine whether large crabs are also able to climb the cordgrass present in salt marsh habitats. Because small crabs are commonly observed climbing cordgrass in the marsh, they were not included in this experiment.

We conducted laboratory trials in which individuals (n = 12, mean ± SE = 22.6 ± 0.4 mm CW) were placed in 5-gallon buckets containing sediment to a height of 50 mm with a single dead stalk of *S*. *alterniflora* (mean ± SE = 1.0 ± 0.4 mm basal stem diameter) placed upright in the center of the bucket to simulate the habitat structure available in marsh habitats. Seawater was then added up to a height of 100 mm to simulate a high tide event, and individuals were placed in the bucket. Crabs in the salt marsh commonly climb both live and dead *S*. *alterniflora* stalks, which provide similar support to crabs. Dead stalks were used in this microcosm experiment to avoid any potential wilting.

Behavior (climbing/not climbing habitat structure) was observed hourly for 6 hours (the length of a high tide cycle). If a crab had not demonstrated the ability to climb the habitat structure by the end of the 6 hours, the water was gently disturbed by hand to encourage the crab to climb the stalk of *S*. *alterniflora* and verify its ability to do so.

Next, we studied size-specific predation potential in both mangrove and mixed marsh habitats. Because individuals (CW>15 mm) are not present in mixed marshes, small individuals (8–15 mm CW) were collected from the representative mixed marsh site and large individuals (18–25 mm CW) were collected from the representative mangrove site for tethering at both habitats. We brought *A*. *pisonii* from both size classes into the laboratory and affixed monofilament tethers (45 cm of 10 lb test) by tying a loop in the monofilament and attaching this loop to the back of the carapace using cyanoacrylate glue. Crabs were maintained in individual aquaria overnight and observed the following morning to ensure that tethers remained secure. Crabs were then transported to the field at low tide. Tethers were attached to mangrove roots or dead *S*. *alterniflora* stalks above the low tide line such that individuals could access the water to stay moist at low tide and exit the water at high tide. Individuals were left in the field for 24 hours and then checked for survival as well as any indications of predation (e.g. pieces of carapace remaining on tether) during low tide. This tethering protocol was repeated four times in each habitat for a total of 149 individuals (mangrove n = 78, mixed marsh n = 71). Because the two habitat types have different physical and structural characteristics, we avoided differences in tethering artifacts between habitats (see review by [38]) by restricting our analyses to comparisons of predation on the two size classes within each habitat using separate generalized linear models with a binomial distribution.

## Results

### Latitudinal life history trends

**Fig 2:**
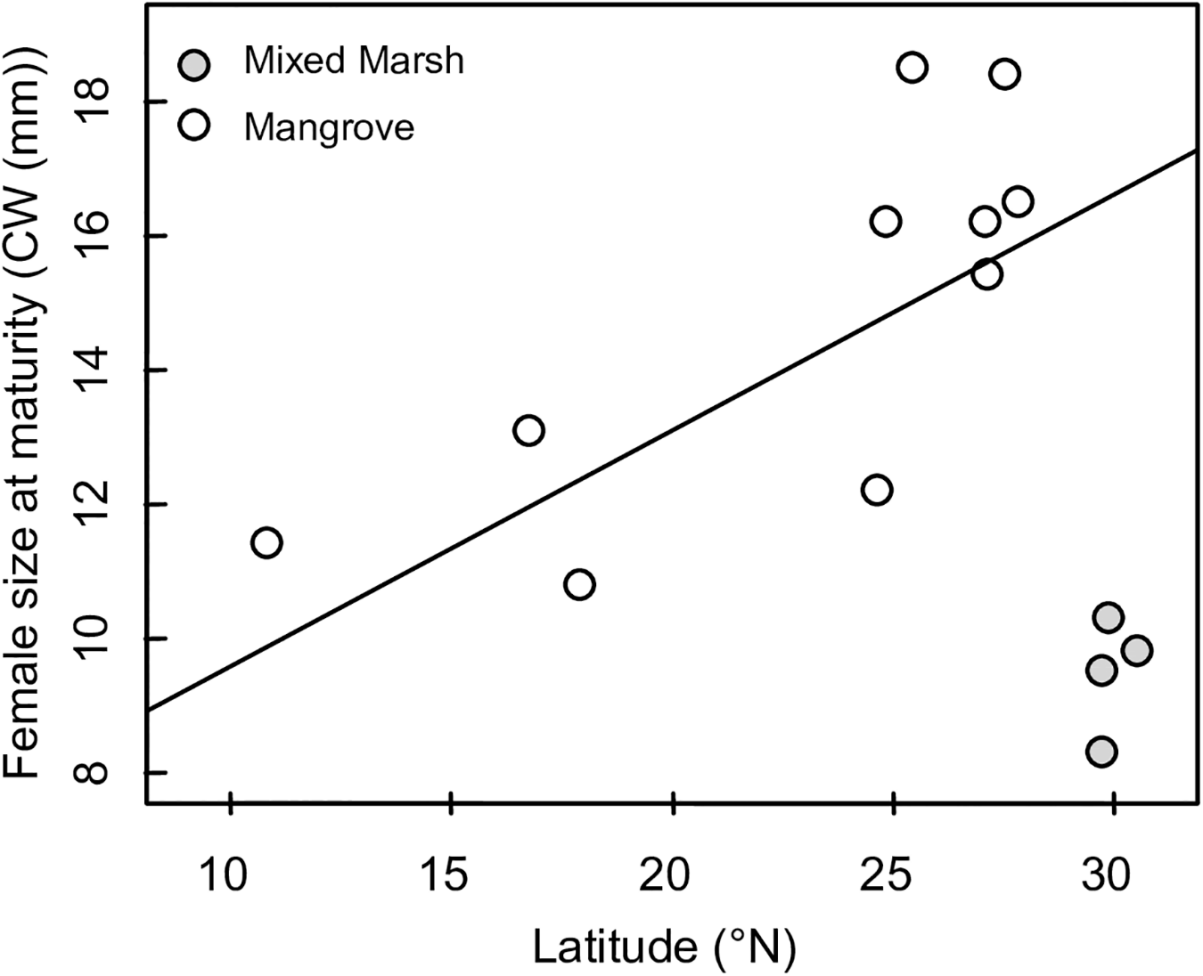
Latitudinal trends in female size at maturity in *A*. *pisonii* in both native and novel habitats.

Female size at maturity increased significantly with latitude, but this trend was countered by the species’ range expansion into novel marsh habitats, as size at maturity was smaller in marshes at the highest latitudes of the species range than in any of the mangrove habitats surveyed (GLM, Latitude: mean slope ± SE = 0.373 ± 0.097, P = 0.003; Habitat: parameter estimate ± SE = 7.986 ± 1.202, P<0.001, Fig 2). Similarly, the average size of ovigerous females increased with latitude (GLM, Latitude: mean slope ± SE = 0.312 ± 0.046, P<0.001), but this trend was reversed at the highest latitudes of the species’ range, where gravid individuals were significantly smaller in novel marsh habitats than native mangrove habitats (GLM, Habitat: parameter estimate ± SE = 8.358 ± 0.313, P<0.001).

### Body size distributions and reproductive effort at representative sites

**Fig 3:**
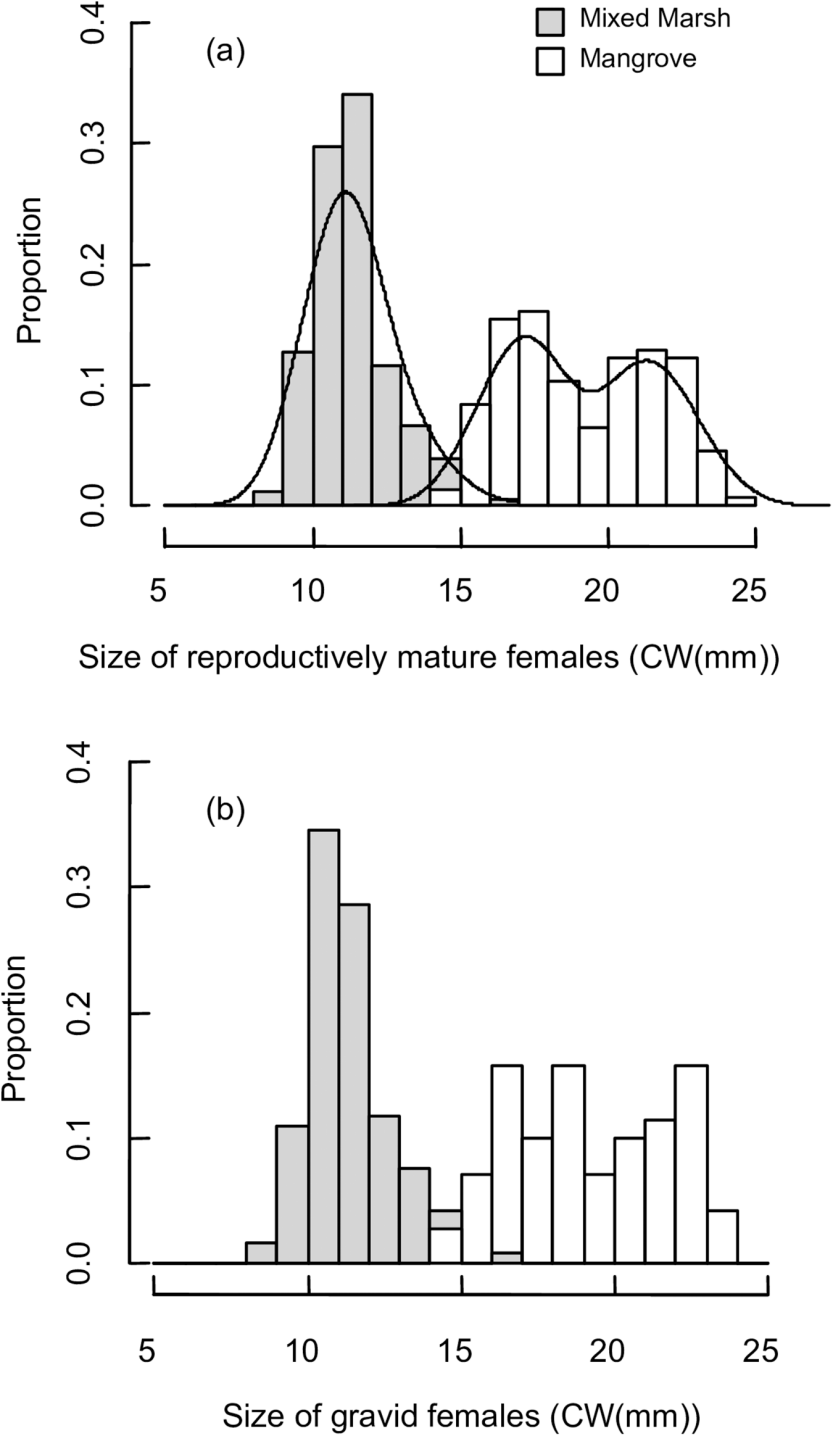
Body size distribution patterns. Includes all *Aratus pisonii* (A) females larger than the site-specific size at maturity and (B) gravid females from June-August 2013. Density curves are included in (A) to highlight modality differences between habitats.

Female size at maturity occurred at 8.9 mm CW in the representative mixed marsh habitat and at 14.4 mm CW in the representative mangrove habitat (Figs 2 and 3). In the marsh, the body size distribution of all females larger than the site-specific size at maturity was unimodal (Hartigan’s dip test, D = 0.031, P = 0.227, Fig 3(A)), while body size distribution of these females was bimodal in the mangroves (Hartigan’s dip test, D = 0.046, P = 0.016, Fig 3(A)). The mean size of gravid females was smaller in the mixed marsh habitat (mean ± SD = 11.4 ± 1.3 mm CW, n = 119) than the mangrove habitat (mean ± SD = 19.2 ± 2.6 mm CW, n = 70) (Wilcoxon rank-sum test, p< 0.001, Fig 3(B)). Additionally, gravid females in the mangrove population displayed 3.53x more variation in body size than those in the mixed marsh population (Fisher’s F-test, p<0.001, Fig 3(B)).

**Fig 4:**
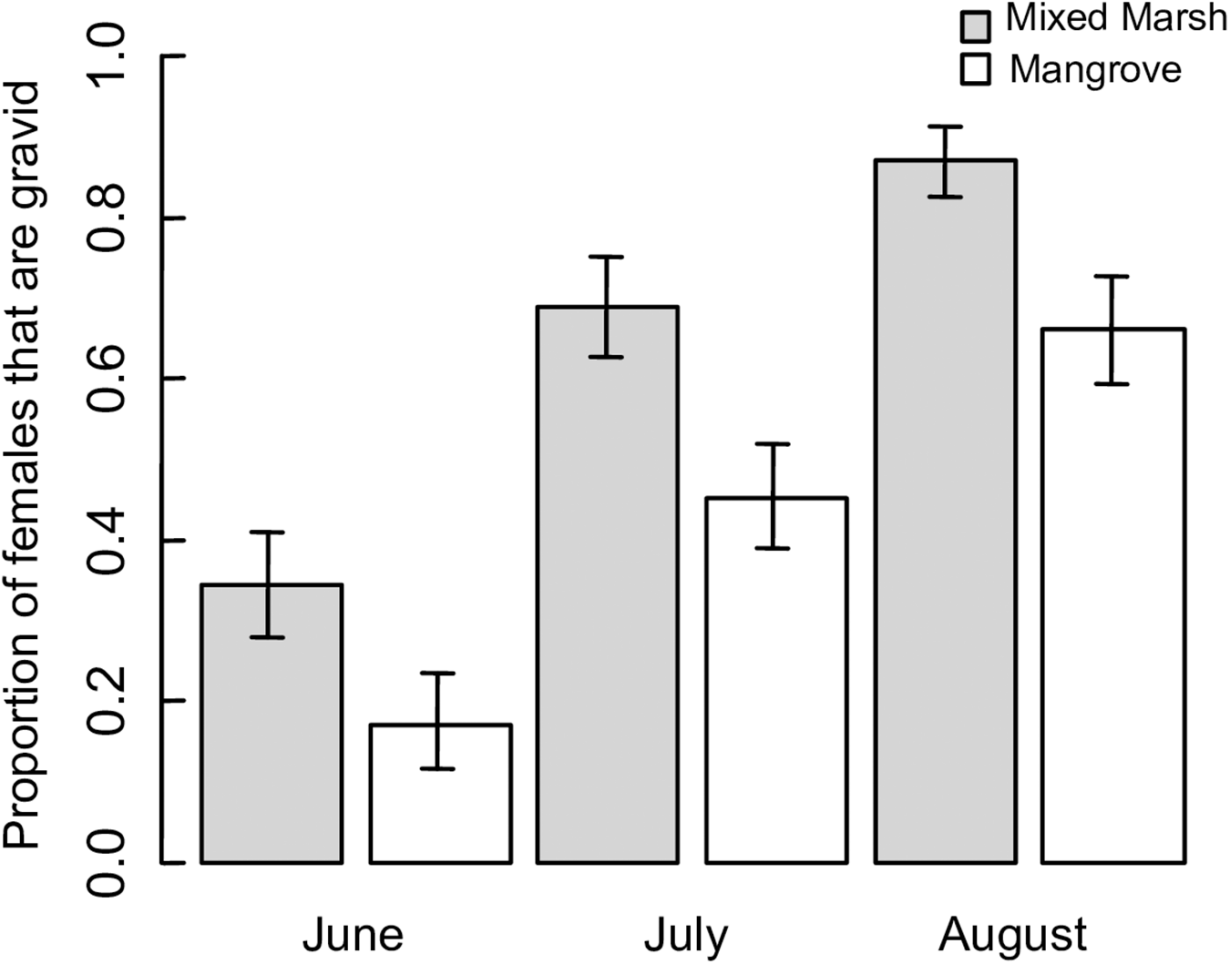
Proportion of gravid females. Proportion of ovigerous females in each *Aratus pisonii* population throughout the survey period. Bars represent standard error.

Although the proportion of females that were gravid increased at both sites over the monthly summer sampling dates, it was consistently higher in the population from the mixed marsh habitat than the population from the mangrove habitat (GLM, Month: P<0.001; Site: parameter estimate ± SD = -1.05 ± 0.25, P<0.001, Fig 4).

### Maternal investment, offspring quality, and fecundity at representative sites

**Fig 5:**
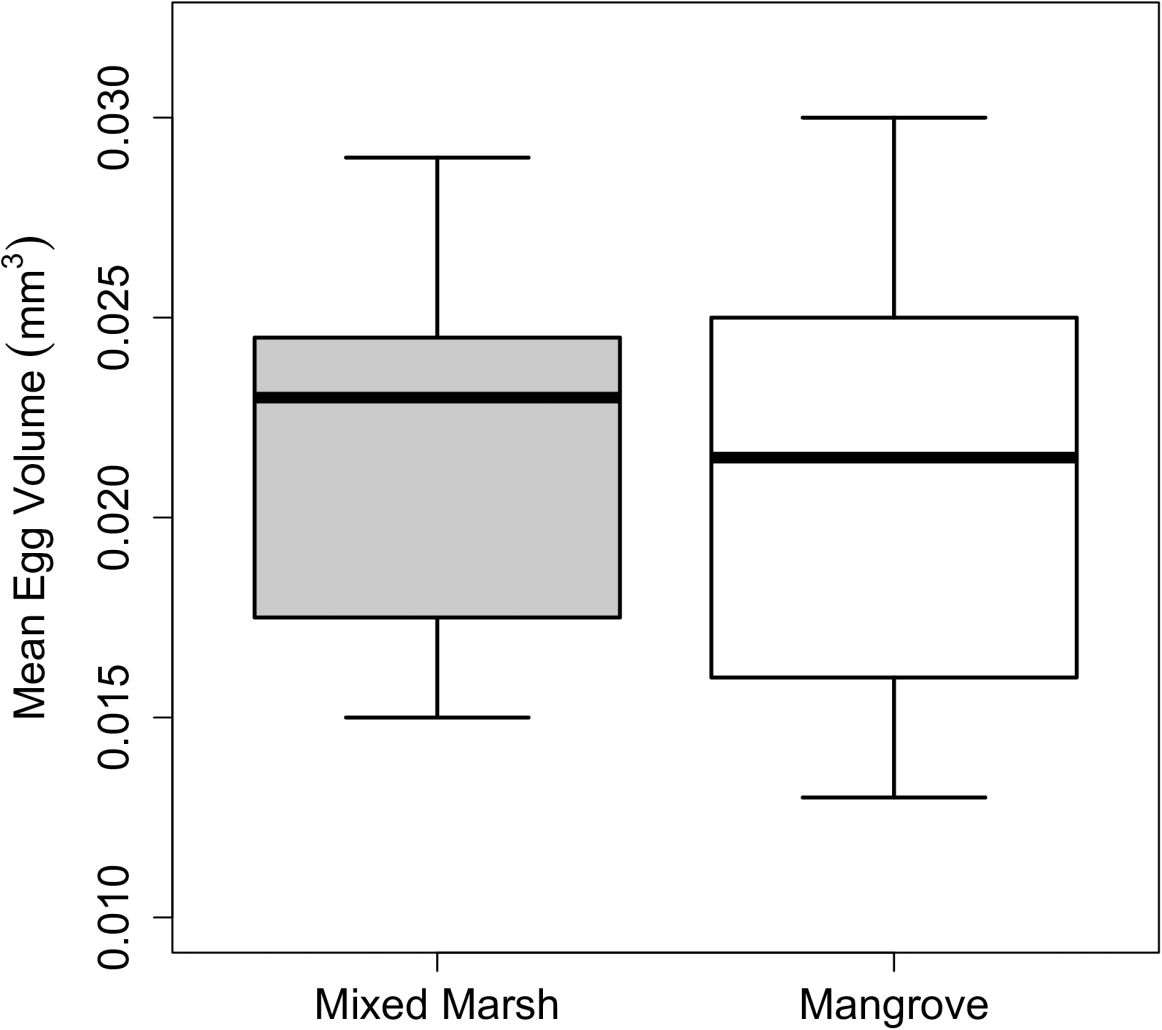
Mean *Aratus pisonii* egg volume (mm^3^) of recently extruded, non-eyed eggs. Bars represent standard error.

**Fig 6:**
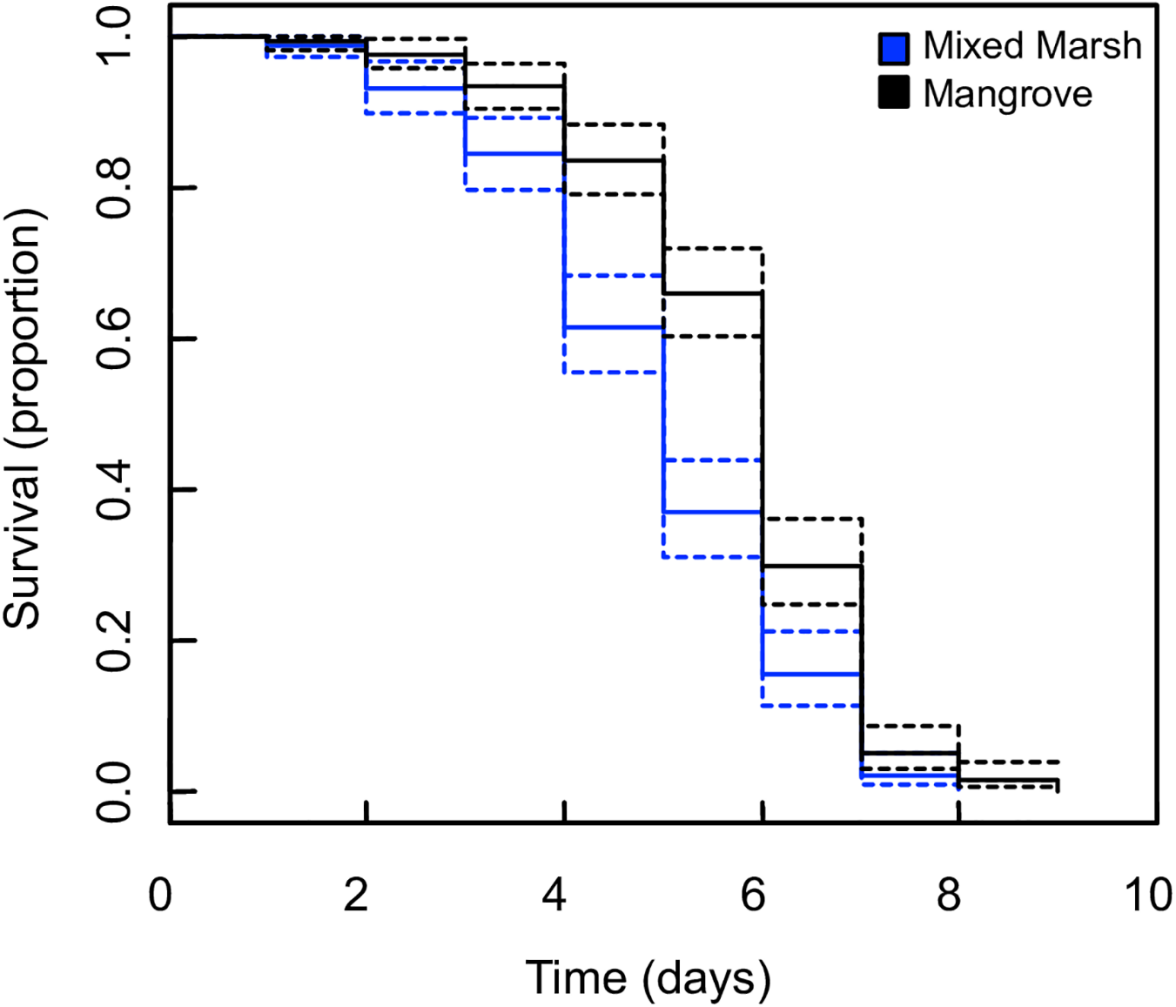
Comparison of *Aratus pisonii* larval survival. Kaplan-Meier curves show *A*. *pisonii* larval survival (i.e., starvation resistance in days) as a function of maternal habitat. Solid lines represent mean survival and dashed lines represent 95% confidence intervals.

Initial maternal investment in eggs, as measured by mean volume of non-eyed eggs, was not impacted by maternal habitat (GLM, mean slope ± SE = 0.001 ± 0.002, P = 0.759, Fig 5). Similarly, individual egg weight (mean for both habitat types = 0.00729 mg), as measured by the slope of the regression between egg count and weight, did not differ between habitat types (GLM, mean slope ± SE = 0.063 ± 0.065, P = 0.343). Larval starvation resistance was significantly impacted by habitat type, with offspring from mothers in the mangroves demonstrating enhanced starvation resistance compared to those from mothers in the mixed marsh (mixed effect Cox model, Maternal habitat: Coefficient ± SE = 0.855 ± 0.300, P = 0.004, Fig 6).

**Fig 7:**
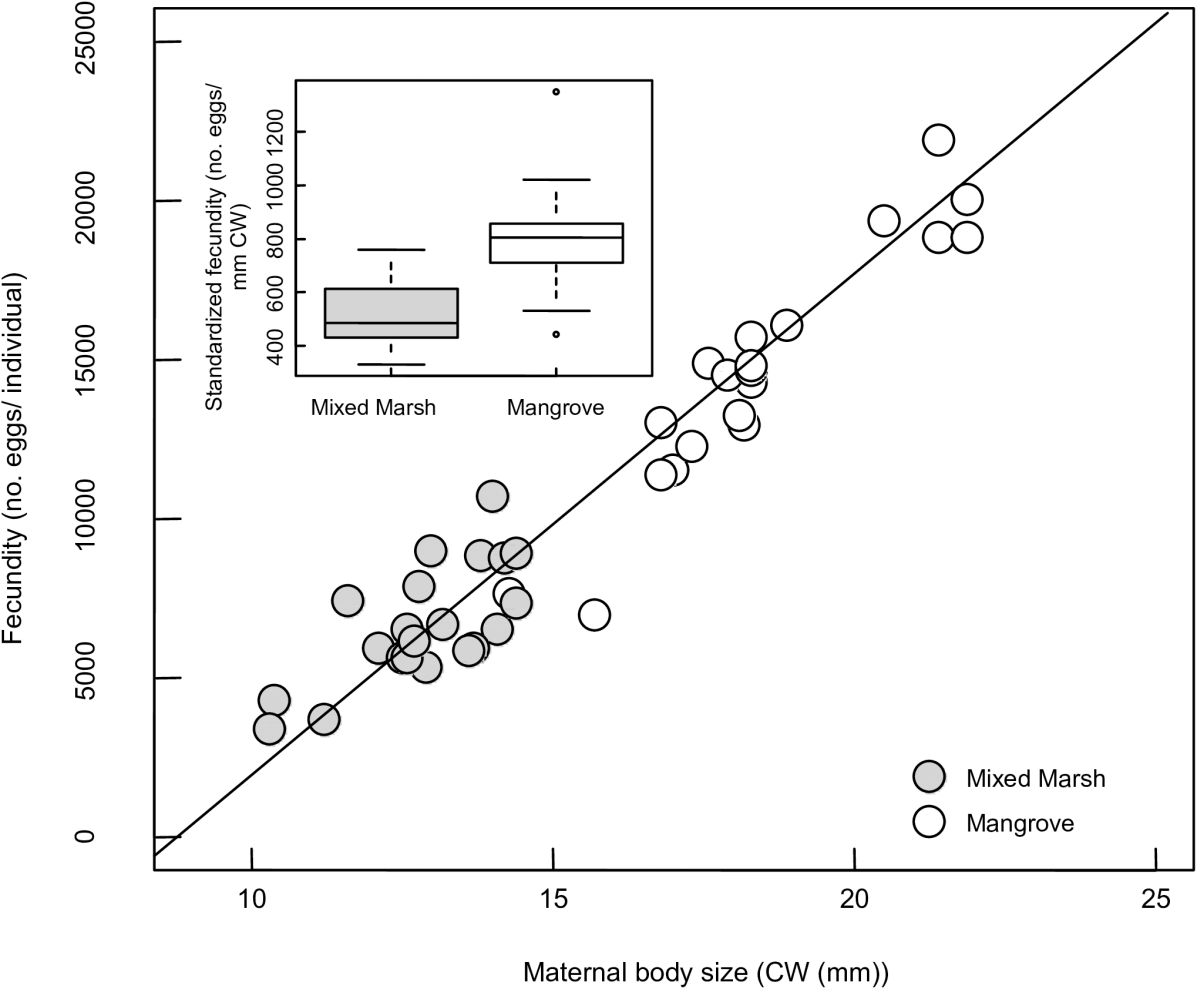
Relationship between maternal carapace width (mm) and fecundity (number of eggs) in *Aratus pisonii*. Inset shows a comparison of standardized fecundity (number of eggs per mm of CW) between habitats.

Fecundity increased significantly with body size but was not explicitly impacted by habitat type (GLM, Maternal body size: mean slope ± SE = 1754.7 ± 191.3, P<0.001; Habitat: parameter estimate ± SE = 1394.0 ± 1255.3, P = 0.274, Fig 7). However, due to differences in maternal body size, fecundity was higher in the mangroves (mean no. eggs/ individual ± SE = 15215.9 ± 1027.6, Fig 7) than in the mixed marsh (mean no. eggs/ individual ± SE = 6649.7 ± 404.9, Fig 7).

### Predator avoidance capability and comparison of size-specific predation potential

**Fig 8:**
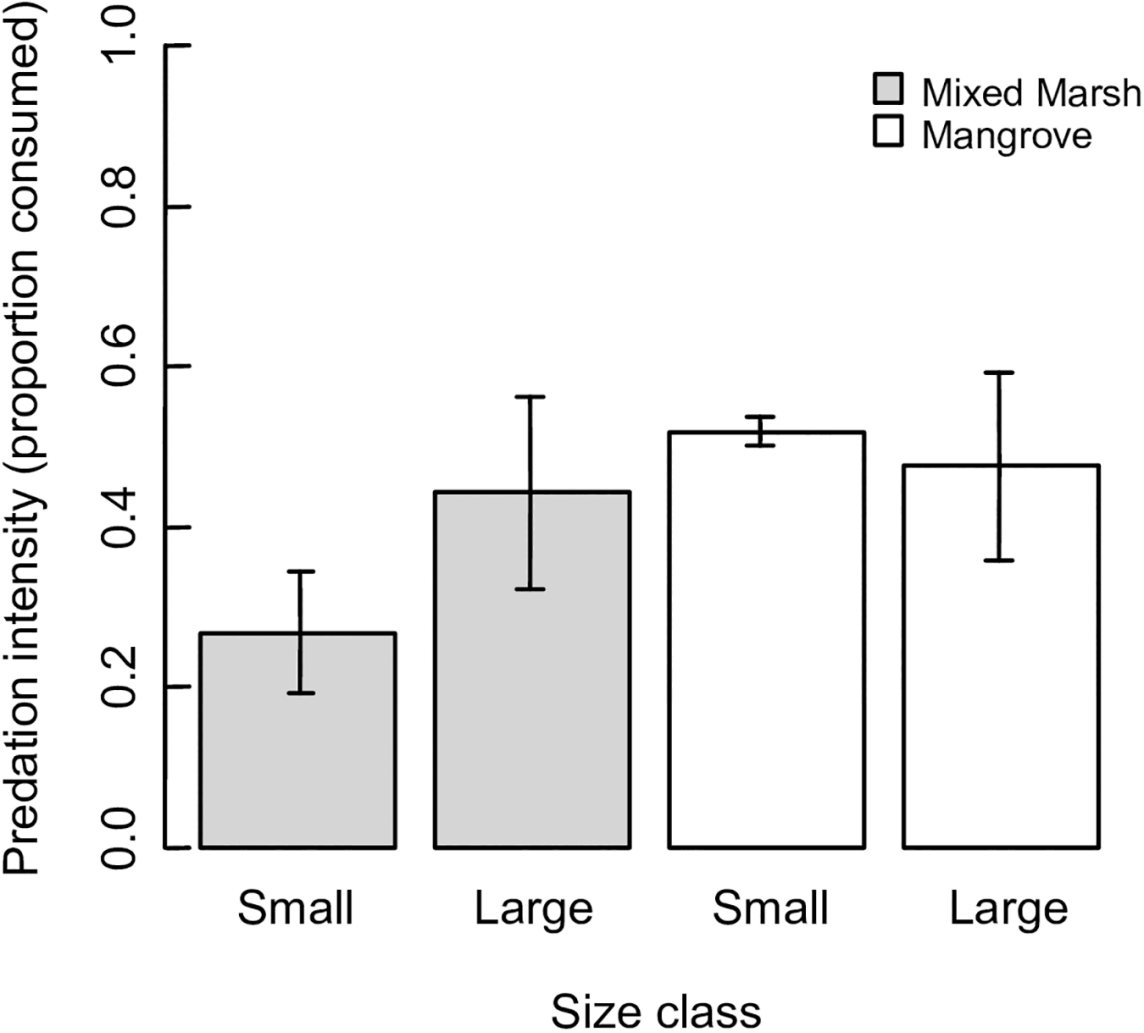
Size-specific predation pressure on individuals of *Aratus pisonii* from native mangrove and novel mixed marsh sites. Bars represent standard error.

All individuals demonstrated the ability to climb *S*. *alterniflora* for predator evasion. Field tethering results indicate that predation potential is nearly twice as high on large crabs than small crabs in novel marsh vegetation (GLM, mean slope ± SE = 0.860 ± 0.501, P = 0.086, Fig 8), although this was only marginally significant, potentially due to insufficient replication. By contrast, tethering experiments revealed no differences in size-selective predation in native mangrove habitats (GLM, mean slope ± SE = 0.041 ± 0.451, P = 0.927, Fig 8).

## Discussion

Our study demonstrates that there is significant variation in key life history traits throughout the current geographic distribution of the mangrove tree crab *Aratus pisonii*. In accordance with traditional biogeographic patterns (i.e. Bergmann’s clines), intraspecific body size, including both size at maturity and average size of reproductive females, increased with latitude within native mangrove habitats. However, this Bergmann’s cline deteriorated in mixed marsh and pure marsh sites at the northernmost latitudes of the species’ distribution. On average, gravid females in marshes at the highest latitudes of the species’ range were just 60% as large as those in the mangroves at lower latitudes. Although it is possible that our method of establishing size at maturity may underestimate intraspecific plasticity within a site, intraspecific body size, including both size at maturity and average size of reproductive females, clearly differed between habitats. This life history pattern suggests that *A*. *pisonii* at the range edge display accelerated sexual maturation, as has been seen in a number of other species expanding their range [39–41].

Although decreased size at maturity at the range edge is consistent with range expansion predictions [15–17], it is an unusual contrast to the Bergmann’s cline demonstrated by *A*. *pisonii* throughout its native range. This contrast is likely due to habitat-specific differences associated with *A*. *pisonii*’s range expansion into novel marsh habitat. Organisms at the range edge often face selective pressures that are unique compared to those experienced by individuals within the core of the species’ range [15–17, 42]. Just as gradients in biotic and abiotic interactions underlie Bergmann’s clines, habitat-specific differences in these elements may also influence life history traits such as body size and size at maturity [17].

Factors such as temperature [43–44], resource availability [23], and predation pressure [27, 45] have been shown to impact life history characteristics. Terrestrial multivoltine arthropods, such as the species described here, generally adhere to the temperature-size rule [22]. The temperature-size rule describes the phenotypically plastic relationship between body size at different developmental stages (e.g. reproductive maturity) and rearing temperature [46]. As a result of this phenotypic response to temperature during ontogeny, individuals reared at low temperatures delay maturity, reaching maturity at a larger body size and growing to be larger overall than conspecifics reared at warmer temperatures [43–44].

Although we did not explicitly compare temperature profiles from the two habitats, the structural complexity of mangrove forests provides numerous shady microhabitats that are lacking in more structurally simplistic marsh habitats. It is well-established that forest canopies maintain lower temperature maximums than contiguous forest gaps or grassy habitats ([47] and references therein), and the temperature profile of red mangrove trees measured in late summer demonstrates that the underside of red mangrove roots is 6.84°C cooler than the top of the roots [30]. Thus, individuals with access to the shaded mangrove understory may experience lower maximum temperatures than their conspecifics in less structurally complex salt marsh habitats. Consequently, our results are consistent with those predicted by the temperature-size rule after incorporating habitat-specific differences within its framework.

It should be noted that interpretation of the temperature-size rule in this scenario is inherently complicated by habitat-specific differences in resource quality and availability. For example, diet quality has been shown to reverse the temperature-size rule in insects: in laboratory experiments, individuals reared on a high quality diet followed the temperature-size rule, while those reared on a low quality diet showed a reversed response, with smaller final sizes at higher temperatures [23]. Although there are no studies in the literature detailing the diet of *A*. *pisonii* in the marsh, their omnivorous diet in native mangrove habitats is well established. In native habitats, mangrove leaves comprise a core component of *A*. *pisonii*’s diet [48]. In addition, individuals commonly consume small prey items such as insects and juvenile conspecifics; consumption of animal material is tightly linked to the fitness and physiological condition of the species [49].

Conversely, mangrove leaves are scarce or entirely absent in novel marsh and mixed marsh sites, forcing individuals to consume alternative food sources. Although little is known about the diet of *A*. *pisonii* in the marsh, a number of scenarios are possible. First, individuals in the marsh may consume a diet containing less animal material or poorer quality vegetation than those in the mangroves. In this case, the crabs in the marsh would be expected to have a slower growth rate than their conspecifics in the mangroves. Alternatively, crabs in the marsh may consume more animal material than those in the mangroves, which would be expected to increase their physiological condition and growth rate [49]. In addition, the quality of the marsh diet may influence whether individuals there display growth patterns consistent with or in opposition to the temperature-size rule (e.g. [23]). Thus, habitat-specific differences in consumption of small prey items such as insects, juvenile conspecifics, or other crustaceans may influence the strength of the relationship between temperature and body size in these two habitats (e.g. [23]). Further studies are needed to tease apart the relative contribution of these mechanisms.

Finally, predation pressure has also been shown to drive differences in both size at maturity and overall body size via phenotypic or genetic processes. For instance, elevated predation intensity from natural predators or commercial harvesting can drive declines in body size at reproductive maturity (e.g. [27, 45] but see [50] for an exception). Our tethering trials indicated that predation potential on *A*. *pisonii* was almost twice as high on large individuals than small individuals in the representative mixed marsh habitat. Conversely, there was no evidence of size-specific predation in the representative mangrove habitat. Because large crabs are not present in salt marsh habitats, all of the large crabs used in the tethering trials were collected from mangrove habitats. Although we previously established that large crabs are capable of climbing cordgrass in the salt marsh, individual inexperience in the salt marsh may have contributed to the increased predation on large individuals. Additionally, habitat may influence prey palatability, and predator preferences may have been impacted by the inclusion of only small crabs collected from the salt marsh in the tethering study. Finally, predator community composition may differ between the two habitat types, which could also impact predation patterns. Nevertheless, even the slight size-specific predation discrepancy noted between large and small individuals in novel habitats could drive the observed decrease in body size and size at reproduction (e.g. [27]). Thus, the life history patterns associated with *A*. *pisonii*’s range expansion are consistent with multiple mechanisms presented here, and we are currently unable to tease apart the relative influence of these explanations.

Variation in size at maturity between habitats was also accompanied by differences in the population structure of reproductive females, which clearly contrasted between native and novel habitats. Specifically, the body size distribution of reproductive females was bimodal in the mangroves and unimodal in the mixed marsh habitat. These distributions suggest that two cohorts (each represented by a peak in body size distribution) are reproducing simultaneously in the population from the native mangrove habitat, while in the marsh population, either a single cohort is reproducing or reduced growth increments prevent the identification of discrete size/age classes. From our data, it is not evident whether differences in the size of ovigerous females reflect true differences in body size at a specific age or merely differences in body size at a particular life stage. Regrettably, a reliable technique to directly determine age in crustaceans does not currently exist [51–52]. A method of direct aging using growth rings in the eyestalks has recently been proposed for cold-water crustaceans [53], but its utility in aging shorter lived, warm-water species such as *A*. *pisonii* requires further examination [53].

In addition to the observed differences in population structure and female size at maturity in mangrove and marsh habitats, fecundity increased significantly with maternal body size, a trait commonly observed in crustaceans (e.g. [54]). Differences in the size of reproductive females in the two habitats therefore resulted in a higher mean nonstandardized fecundity (mean no. eggs/ individual) in native mangrove habitats than novel marsh habitats. Initial maternal investment in individual eggs, as measured by the mean egg volume and egg weight of recently extruded eggs, was not impacted by maternal habitat. However, offspring quality, as measured by larval starvation resistance, was improved in native mangrove habitats compared to novel salt marsh habitats. Because there was no relationship between egg volume or weight and maternal habitat, these differences in starvation resistance likely reflect differences in the quality, but not the quantity, of egg provisioning, which could result from habitat-wide differences in maternal physiological condition or previous reproductive investment. These differences in offspring quality may have enduring consequences for the survival and persistence of this species in novel habitats.

## Conclusion

In this study, we demonstrate that a climate change-induced range expansion into a novel habitat overrules the Bergmann’s cline demonstrated by the species within its native habitat, potentially due to differences in predation pressure or environmental conditions in the native and novel habitats. The deterioration of this biogeographic body size pattern has a number of implications for fecundity and offspring quality in novel habitats at the edge of the species’ range. As climate change advances, spatial mismatches between species distributions will continue to reshuffle biological communities, leading to novel communities and unique plant-animal interactions [12–14]. Understanding the potential for life history changes associated with range expansions, as well as the mechanisms underlying these life history alterations, is critical for the prediction of key demographic processes [17, 55].
